# Religiosity, religion values, and science values in Northern Ireland and the Republic of Ireland

**DOI:** 10.1371/journal.pone.0331205

**Published:** 2025-10-08

**Authors:** Hannah J. Kramer, Niamh McLoughlin, Kathleen H. Corriveau, Jocelyn B. Dautel

**Affiliations:** 1 School of Psychology, Queen’s University Belfast, Belfast, Northern Ireland, United Kingdom; 2 Department of Psychology, University of Wisconsin, Madison, Wisconsin, United States of America; 3 Wheelock College of Education and Human Development, Boston University, Boston, Massachusetts, United States of America; 4 MIT Blueprint Labs, Massachusetts Institute of Technology, Boston, Massachusetts, United States of America; University of Cambridge, UNITED KINGDOM OF GREAT BRITAIN AND NORTHERN IRELAND

## Abstract

We investigate the relation between religion and science in Northern Ireland and the Republic of Ireland. That is, we test the extent to which religiosity and valuing religion correspond with valuing science. We explore two contexts where religion and science are integral parts of society—Northern Ireland and the Republic of Ireland. As part of a larger study, participants (*N* = 739 adults living in Northern Ireland or the Republic of Ireland) reported their level of religiosity as well as responded to a survey that measured how much they value religion and science. In both contexts, religion and science were negatively related. In other words, participants who reported being more religious or who valued religion more tended to value science less. Findings are discussed in relation to the value of taking a global approach to studying religion and science as well as how this type of relation may contribute to polarization.

## Introduction

Religion and science draw on different mechanisms to provide explanations for the same phenomena. For example, the universe was created by a massive explosion or by a divine agent; humans evolved over time from other species or were created in the image of an all-powerful being. Religion and science can also appear to differ in their search for evidence, with science being more oriented towards the evidential process and religion focusing on the evidential outcome. They are further distinguishable by their perceptions of truth—whereas science aims to discover the (sometimes changing) truth, religion provides the (always stable) truth [1]. Thus, at least intuitively, it may feel as though religion and science are at odds [2]. Do people’s actual experiences of religion and science work in this way? We explore relations between religiosity, valuing religion, and valuing science to understand whether these attitudes are opposing (i.e., if you are religious then you cannot value science) or if it is possible for the same person to hold competing values (i.e., a person can appreciate both religion and science simultaneously). Assessing this connection can provide better insight into how people’s experiences with these two domains can fuel polarization within and across societies.

Importantly, it is unlikely that the relation between religion and science will be consistent around the world [3]. Here, we consider how context might shape the extent to which beliefs about religion and science covary with one another. In particular, we focus our investigation on Northern Ireland and the Republic of Ireland because religion is embedded in both societies (both historically and in the present day). At the same time, however, both regions of the world have made significant investments in science and technology in recent decades, such as the growth of multinational tech sectors in Republic of Ireland and the expansion of cybersecurity and health technology industries in Northern Ireland [4]. Although this trend aligns with broader global shifts toward scientific and technological innovation, examining how these developments intersect with locally specific religious and political dynamics offers a unique lens on the relation between science and religion. These contexts are of further interest because whereas Northern Ireland has a recent history of violent conflict (in part surrounding ethno-religious identity), the civil unrest did not directly affect the Republic of Ireland to the same extent. Thus, a comparison of these two societies offers a unique opportunity to understand whether tension at a societal level within one domain (here, religion) could be related to how individuals within the society not only value religion but also devalue another domain (here, science).

It may be more than intuition that religion and science are incompatible. For example, religious individuals in the United States prefer that scientists are not involved in policy decisions that involve moral issues (i.e., global warming, stem cell research; [1]). Academic scientists also tend to be less religious than the general American public [5,6]. Moreover, scientists raised in religious households or whose beliefs changed from being religious to nonreligious judge that there is conflict between religion and science [7,5]. Chan [8] further documented that across 52 countries religiosity was negatively related to science orientation (e.g., people who were more religious viewed scientists as less trustworthy). Thus, being more scientifically leaning may run counter to holding a religious outlook.

Despite work showing that religious and scientific views are conflicting (i.e., negatively related), studies have also found individuals can hold “co-existing” belief systems. The relation between religious and scientific beliefs can be null or people sometimes use religious and scientific explanations in tandem to understand the world (e.g., [3,9]). Legare and colleagues [10] lay out several examples showing that the co-existence of natural and supernatural beliefs are common in children’s and adults’ thinking. For instance, Legare and Gelman [11] demonstrated that children and adults in South Africa rely on both natural (i.e., biological) and supernatural (i.e., bewitchment) causes to explain serious illnesses (e.g., believing that AIDS is caused by blood mixing but also by dissatisfied ancestors). Across development and cultural contexts, individuals incorporate both biological and religious ideas into their understanding of death (e.g., [12,13]). College students in the United States also provide similar types of justifications when explaining their religious and scientific beliefs [14]. Moreover, a longitudinal study of undergraduates across the United States revealed that students in their freshman year were more likely to view religion and science as independent or in collaboration more so than in conflict; this perspective further increased between students’ first and third years of college [15]. Therefore, science and religion do not have to be opposing; rather, it is common for them to share space in people’s minds.

As alluded to previously, whether science and religion are opposing may be an outcome of the greater societal religious and scientific context. Although Chan [8,16] found an overall negative relation between religiosity and science orientation, the relation varied by country (in Thailand, Lebanon, Philippines, Libya, Nigeria, Ghana, South Africa, Taiwan, Pakistan, Iraq, and Jordan there was a positive relation between religiosity and confidence in science). Payir and colleagues [3] documented a negative relation between religiosity and science values in China and the United States. In other words, people who valued science were less likely to be religious. Still, Payir and colleagues [3] also documented that in Iran the link between religiosity and science values was null. Taken together, these results provide evidence that as religiosity increases, valuing science does not by default decrease.

In our use of the term, valuing science refers to broad attitude of perceiving science as important, beneficial, and relevant for understanding and improving the world, whereas valuing religion refers to the broad attitude of perceiving religion as meaningful, morally significant, and personally or socially important. These are not necessarily mutually exclusive domains, but their relation may be at least partially explained by broad societal differences. Indeed, Payir and colleagues [3] described the difference in relations among the US, China, and Iran as differences in the embeddedness of religion within broader society. In Iran, religion is particularly integrated across society (e.g., within laws and schools) whereas, in China and the US, stricter divides are maintained between religious and other public institutions. Thus, when studying the relation between religion and science it is important to consider context, including both individual beliefs and the societal structures in which those beliefs are embedded.

The goal of the current work was to expand inquiry on relations between religion and science to two new socio-political locations: Northern Ireland and the Republic of Ireland. Northern Ireland and the Republic of Ireland are intriguing places to test relations between religion and science because they are both Western societies where individuals hold strong religious beliefs [17,18,19]. Moreover, like Iran, religion is embedded within both societies. In particular, there is no formal separation of church and state on the isle of Ireland. For example, whereas public funding is not available for schools with religious affiliation in US, in Northern Ireland and the Republic of Ireland religious education is required in the curriculum [20,21]. In addition, at least during the primary school years, children often learn about religion and science from the same teacher. Thus, valuing religion and science is simultaneously modeled within the same person for children during their development. Science is also prominent in these contexts. Indeed, both Northern Ireland and the Republic of Ireland are growing technological hubs. The Republic of Ireland is sometimes referred to as the Silicon Valley of Europe as it is the European home for Google, Facebook, and Linkedin. This increased focus on technology has also begun to bleed into Northern Ireland which accounts for 17% of the United Kingdom’s high-growth technology firms, especially cybersecurity and healthcare [4]. Thus, both religion and science appear to be central within Northern Ireland and the Republic of Ireland, potentially giving the appropriate sociopolitical conditions for a null or positive relation between religion and science.

Despite their similarities, an important distinction can be made between Northern Ireland and the Republic of Ireland, Northern Ireland is a post-conflict society where religious affiliation serves as a key marker of political and national identity [22]. Although the 1998 Belfast/Good Friday Agreement formally ended large-scale violence more than two decades ago, tensions and religious and political divisions between the two groups, most commonly labelled Catholic and Protestant, remain deeply embedded in social life. For example, Catholics and Protestants often attend different schools, live in separate neighborhoods, play different sports, consume different media, and engage with distinct sets of symbolic markers that reinforce group boundaries and cultural identity [23]. In contrast, the Republic of Ireland, historically shaped by a relatively homogeneous Catholic population, has not experienced the same extent of ethno-religious conflict in recent decades. This difference may contribute to a less polarized relationship between religion and science in everyday life, as religion plays a less overt role in shaping political identity and institutional trust.

These historical and identity-based divisions also help explain the differing power dynamics and social roles of religion across the two contexts. In Northern Ireland, religion is intertwined with political institutions, with religious leaders and parties exerting influence over education, governance, and public discourse [24,25]. For instance, several members of the Democratic Unionist Party (DUP) have publicly rejected scientific consensus on evolution and climate change [26], illustrating how religious and political alignment can contribute to public skepticism toward scientific authority. In contrast, the Republic of Ireland has become increasingly more secular/extra-institutional, particularly following scandals involving the Catholic Church (Ganiel, 2016) [27]. Although Catholicism remains the dominant cultural identity in the Republic of Ireland, the Church’s formal role in state institutions has declined, creating a sociopolitical environment where science is potentially less likely to be framed in opposition to dominant cultural narratives.

By comparing the relation between religion and science values across the context of Northern Ireland and the Republic of Ireland, we are able to examine how religious identity as intertwined with both cultural and political power dynamics, potentially shaping the relative value of religion, as well as the tension between religion and science.

### Current research

We examined relations between religiosity, religious values, and science values in two new contexts: Northern Ireland and the Republic of Ireland. As part of other projects, we included the measures that we will discuss below in two small, initial pilot studies in Northern Ireland which led to inconsistent results. The first pilot study resulted in a significant negative relation between religiosity and science values and the other pilot yielded null results, but the point estimates in each study did not differ from one another (see S1 Table Pilot Studies 1 and 2). Thus, we conducted a new pre-registered study with a larger sample size to allow for a more accurate representation of the relation between religion and science in Northern Ireland, and to compare with this relation in the Republic of Ireland. This study examines whether and how the relations between religiosity, religious values and science values differ across two culturally similar but socio-politically distinct contexts. Although focused on individual-level endorsement of religious and scientific values, the study is informed by broader societal factors—such as the role of religion in political discourse and historical conflict—that may shape how these values are expressed and related [3].

## Method

### Participants

In Northern Ireland, 411 adults consented to participate and in the Republic of Ireland, 376 participants consented to participate. We originally pre-registered the inclusion of 400 participants in Northern Ireland and 400 participants in the Republic of Ireland. To be included in this study, participants had to be living in either Northern Ireland or the Republic of Ireland. Initially, we attempted to recruit participants who had also been born in that same respective region. About half way through data collection, we decided to remove this inclusion criteria because we were having difficulty recruiting participants. Participants also had to have a 95% approval rating within Prolific. Our Republic of Ireland sample is slightly smaller than planned because we had more difficulty recruiting participants meeting this criteria than we had expected.

There were instances where participants reported the same Prolific ID, suggesting that the same participant completed the study twice. We removed the second attempt from each of these participants (i.e., we only kept their first attempt; Northern Ireland: n = 2; Republic of Ireland: n = 5; we did not pre-register this intention). A priori, we determined that we would exclude participants for several reasons: (a) failing or not answering both attention checks (Northern Ireland: n = 6; Republic of Ireland: n = 10); (b) providing a non-normative response to the question at the end of the survey asking what they had done during the study (Northern Ireland: n = 2; Republic of Ireland: n = 2); (c) completing the study in less than 10 min (Northern Ireland: n = 0; Republic of Ireland: n = 0); and (d) reporting that their responses to the survey were not thoughtful or not responding to this question (Northern Ireland: n = 7; Republic of Ireland: n = 5). We also removed participants who did not consent to us posting their data to the Open Science Framework repository (Northern Ireland; n = 4; Republic of Ireland: n = 5; we did not pre-register this intention). Our final sample of 739 gave us 80% power to detect a regression coefficient of small effect (f^2^ =.01) within a linear regression model with eight predictors.

In Northern Ireland (*N* = 390; *M*_age_ = 37.36, *SD*_age_ = 11.62, range: 18–75 years or older; In NI and the ROI, participants reported their age in years from 14 years and older (all participants were over 18 years) to 75 years and older. For the purpose of analyses, we treated participants 75 years and older as 75 years), 39% of participants identified as coming from a Catholic background, 50% from a Protestant background, 5% from both backgrounds, 5% from neither background, and 1% from another background. Participants were also asked two other separate religion questions at the beginning of the study. Because we were interested in ethno-religious background, we also asked a question that was phrased: “In NI, traditionally there have been two major communities. What community background do you come from (e.g., thinking about the background of yourself, your parents, grandparents, or great-grandparents)?” For the first 20 participants, we used different wording: “In NI, traditionally there have been two major communities. What community background do you come from?” 48 % of participants identified as a woman, 51% as a man, and 1% as another gender. Participants were 2% Asian, 96% white, and 2% identified as another ethnicity or belonged to multiple racial/ethnic groups (1 participant chose not to answer this question). When asked where they fell on a ladder representing their relative socio-economic standing (1 = at the bottom of the ladder or least well off to 10: At the top of the ladder or most well off; i.e., their perceived socio-economic status), 32% reported falling below the midpoints (i.e., 1, 2, 3, 4), 44% at the midpoints (i.e., 5, 6), and 28% above the midpoints (i.e., 7, 8, 9, 10; one participant chose not to answer this question). In terms of education, 63% of participants reported that they had an undergraduate degree or higher (one participant chose not to answer this question). A small number of participants in Northern Ireland and the Republic of Ireland selected the “other” education option, but we were able to recode them into existing categories based on their explanation of “other.”

In the Republic of Ireland (*N* = 349; *M*_age_ = 35.17, *SD*_age_ = 10.88, range: 19–75 or older), 78% of participants reported that they came from a Catholic background, 6% from a Protestant background, 3% from both, 6% from neither, and 8% from another background. Participants were also asked two other separate religion questions at the beginning of the study. Because for this study we were interested in ethno-religious background, we also asked a question that was phrased: “What background do you come from (e.g., thinking about the background of yourself, your parents, grandparents, or great-grandparents)?” For the first 20 participants, we used different wording: “Which of the following best describes you?” 57% of participants identified as a woman, 42% as a man, and 1% as another gender. Participants were 2% Black, 3% Asian, 92% white, and 3% identified as another race/ethnicity or belonged to multiple racial/ethnic groups (1 participant chose not to answer this question). When asked where they fell on a ladder representing where they stood in the country, 20% reported falling below the midpoints, 48% at the midpoints, and 32% above the midpoint (one participant chose not to answer this question). In terms of education, 77% of participants reported that they had an undergraduate degree or higher.

### Procedure

Participants completed measures of religiosity, religious values, and science values. Participants also provided demographic information (e.g., gender, education level, perceived socio-economic status, and race/ethnicity). For the purpose of transparency, we wish to highlight that we had originally pre-registered that we would also explore relations between cultural socialization and participants’ values but determined that this analysis was better suited to a separate paper. Data for this project were collected in collaboration with the Developing Belief Network. Thus, participants responded to several other measures and ultimately there will be data from many other sites (most other sites, however, are not collecting data on religious and science values). Participants were recruited from Prolific. The entire survey took approximately 45 min to an hour and participants received compensation for their participation. Data were collected between December second, 2022 and April 18th, 2023. This study was approved by the ethics boards at Queen’s University Belfast (protocol number: EPS 21_300); protocol number and University College Dublin (protocol number: TMREC_PSY: 2021-15). Participants in this study read an information sheet and then agreed to participate in the study before moving on, documented by a series of check boxes.

### Religiosity

Participants responded to the question: “Compared to adults in your local area (for example, your city or town), how religious do you consider yourself to be?” Participants answered this question on an 11-point scale from 0 (I am less religious than everyone in my local area) to 10 (I am more religious than everyone in my local area) with 5 being the midpoint (About half of the people in my local area are less religious than I am, and about half are more religious than I am).” This self-referential religiosity measure allows for comparison across participants from diverse backgrounds and regions (sample ranges: Northern Ireland = 0–10; Republic of Ireland = 0–10). We acknowledge, however, that this measure does not capture behavioral or ritual aspects of religiosity. Thus, although not pre-registered we decided to also include two additional measures of religiosity to get a more complete picture of participants’ religious practices: We measured the frequency with which participants engaged in public religious practices and private religious practices. Participants reported their responses on a scale from 0 (Never) to 8 (Multiple times a day; sample ranges for Public Religious Practice in Northern Ireland= 0–7, in Republic of Ireland = 0–6; Private Religious Practices in Northern Ireland = 0–8, in Republic of Ireland = 0–8).

### Valuation of science and religion

Following Payir and colleagues [3], participants reported how much they agreed with 22 statements about their valuation of religion (11 items) and science (11 items) on a 5-point scale from “strongly disagree” (1) to “strongly agree” (5). Example items include (“To me, it is important to have a [scientific/religious] outlook in life”; “It is important for children to be raised with a [scientific/religious] outlook in life). Items were presented in a fixed order with science-related items coming before religion-related items. See Table 1 for items and descriptive data. The valuation measures from Payir and colleagues [3] were selected for their cross-cultural utility and were reviewed for relevance in both Northern Ireland and the Republic of Ireland. In Northern Ireland, average science valuation and religious valuation ranged from 1 to 5 across participants; in the Republic of Ireland, average science valuation ranged from 2 to 5 and average religious valuation ranged from 1 to 4.

**Table 1:** Means [and 95% Cis] for the science and religious values survey in Northern Ireland and the Republic of Ireland.

	Northern Ireland	*Republic of Ireland*
Item	Mean [95% CI]	Mean [95% CI]
To me, it is important to have a scientific outlook on life.	4.01 [3.91, 4.11]	4.12 [4.02, 4.22]
*It is not very important to visit a science museum regularly.	2.98 [2.86, 3.09]	3.03 [2.91, 3.16]
*It is not very helpful to discuss scientific matters with other adults.	3.92 [3.82, 4.02]	4.08 [3.98, 4.18]
*It is not very important to read and understand scientific texts.	3.75 [3.64, 3.85]	3.84 [3.72, 3.95]
It is important to be open to guidance of people with scientific expertise.	4.27 [4.20, 4.34]	4.27 [4.19, 4.36]
It is important for children to be raised with a scientific outlook on life.	4.20 [4.13, 4.28]	4.18 [4.10, 4.26]
*It is not very important for children to visit a science museum regularly with their parents.	3.37 [3.25, 3.49]	3.40 [3.28, 3.52]
*It is not very helpful to discuss scientific topics with children.	4.14 [4.05, 4.23]	4.24 [4.15, 4.34]
*It is not very important for children to read and understand scientific texts.	3.92 [3.82, 4.02]	3.98 [3.87, 4.08]
It is important for children be open to the guidance of people with scientific expertise.	4.26 [4.19, 4.33]	4.30 [4.22, 4.38]
I turn to science for answers to key questions in life.	3.62 [3.51, 3.73]	3.76 [3.65, 3.87]
**Science Valuation Score Average**	**3.85 [3.79, 3.91]**	**3.93 [3.86, 3.99]**
To me, it is important to have a religious outlook on life.	2.52 [2.38, 2.66]	2.23 [2.10, 2.36]
*It is not very important to visit a place of worship regularly.	2.32 [2.19, 2.45]	2.12 [2.00, 2.25]
*It is not very helpful to discuss religious matters with other adults.	3.10 [2.98, 3.21]	2.88 [2.75, 3.00]
*It is not very important to read and understand religious texts.	2.84 [2.72, 2.96]	2.56 [2.44, 2.68]
It is important to be open to the guidance of people with religious expertise.	3.09 [2.97, 3.21]	2.78 [2.65, 2.90]
It is important for children to be raised with a religious outlook on life	2.66 [2.54, 2.79]	2.34 [2.22, 2.47]
*It is not very important for children to visit a place of worship regularly with their parents.	2.53 [2.41, 2.66]	2.28 [2.15, 2.41]
*It is not very helpful to discuss religious matters with children.	3.42 [3.31, 3.53]	3.19 [3.07, 3.31]
*It is not very important for children to read and understand religious texts.	2.96 [2.84, 3.08]	2.58 [2.46, 2.70]
It is important for children be open to the guidance of people with religious expertise.	3.17 [3.06, 3.29]	2.84 [2.71, 2.97]
I turn to religion for answers to key questions in life.	2.28 [2.15, 2.42]	1.91 [1.79, 2.03]
**Religious Valuation Score Average**	**2.81 [2.72, 2.90]**	**2.52 [2.43, 2.61]**

*Indicates that the item has been reversed scored prior to analysis.

### Transparency

The current study, including sample size, exclusions, research questions, and analyses were preregistered on the Open Science Framework (https://osf.io/da3jf/?3918a0ae30d64ad79f087bc2aa7fe267); we highlight any place that we strayed from our original plan. De-identified data and analysis code are available on the Open Science Framework (https://osf.io/ctd39/?ce8c570cefd844d09b7bcee7991fa274). Materials are available in the main text of this article.

## Results

Participants responded consistently to items that were meant to tap science values (Northern Ireland: Cronbach’s alpha =.82, 95% CI[.80,.85]; Republic of Ireland: Cronbach’s alpha =.84, 95% CI[.81. 86]) and to those that were included to represent religion values (Northern Ireland: Cronbach’s alpha =.93, 95% CI[.92,.94]; Republic of Ireland: Cronbach’s alpha =.90, 95% CI[.89,.92]). Participants in Northern Ireland and Republic of Ireland did not differ in their science values (Northern Ireland: *M* = 3.85, 95% CI[3.79, 3.91]; Republic of Ireland: *M* = 3.93, 95% CI[3.86, 3.99]; *t*[729.03] = −1.82, *p* =.070, *d* = 0.13) or how frequently they engaged in public religious practices (Northern Ireland: *M* = 1.36, 95% CI[1.19, 1.53]; Republic of Ireland: *M* = 1.20, 95% CI[1.06, 1.34]; *t*[721.28] = 1.40, *p* =.163, *d* = 0.10), but participants in Northern Ireland reported being more religious (Northern Ireland: *M* = 3.02, 95% CI[2.77, 3.27]; Republic of Ireland: *M* = 2.63, 95% CI[2.38, 2.88]; *t*[729.63] = 2.15, *p* =.032, *d* = 0.16), more frequently engaging in private religious practices (Northern Ireland: *M* = 1.81, 95% CI[1.56, 2.06]; Republic of Ireland: *M* = 1.46, 95% CI[1.23, 1.70]; *t*[737] = 2.01, *p* =.045, *d* = 0.15), and valuing religion more than did participants in Republic of Ireland (Northern Ireland: *M* = 2.81, 95% CI[2.72, 2.90]; Republic of Ireland: *M* = 2.52, 95% CI[2.43, 2.61]; *t*[736] = 4.44, *p* <.001, *d* = 0.33). In Northern Ireland and Republic of Ireland, participants reported valuing science more than religion (Northern Ireland: *t*[387] = 16.41, *p* <.001, *d* = 0.83; Republic of Ireland: *t*[348] = 23.39, *p* <.001, *d* = 1.25). Note that inclusion of private and public religious practices throughout the results was not pre-registered and should be interpreted with caution. The pre-registered analyses, however, match the findings that we report here.

**Fig 1:**
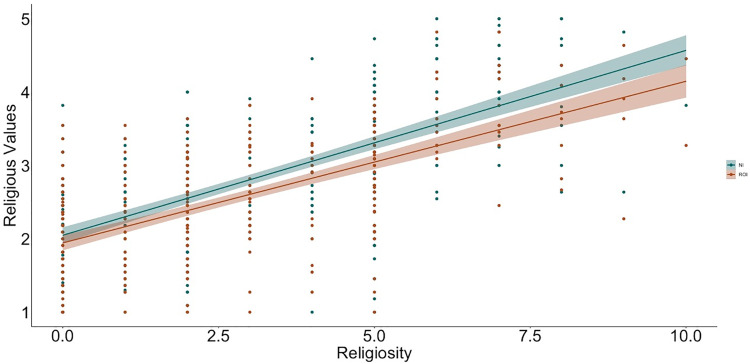
Relation between religiosity and religious values by context (NI, Northern Ireland, ROI, Republic of Ireland).

**Fig 2:**
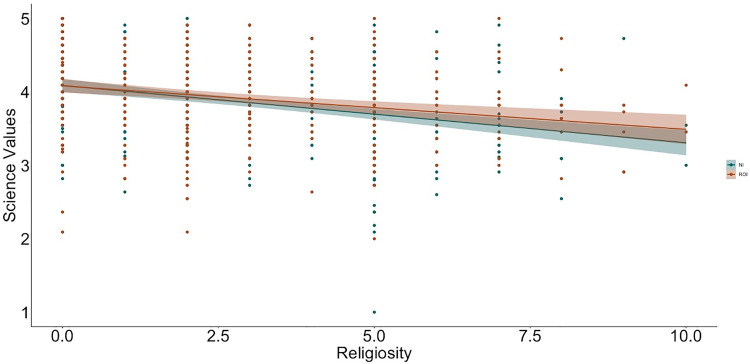
Relation between religiosity and science values by context (NI, Northern Ireland, ROI, Republic of Ireland).

**Fig 3:**
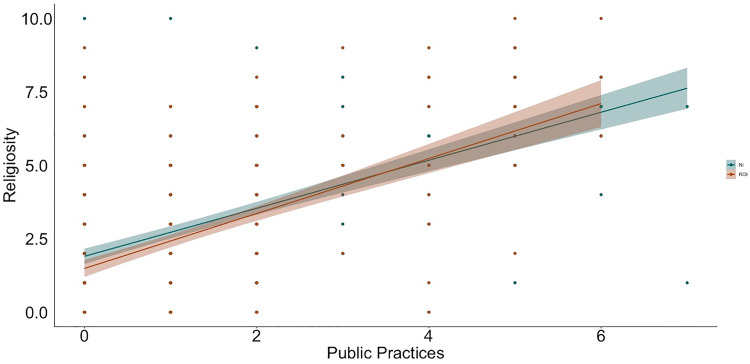
Relation between public practices and religiosity by context (NI = Northern Ireland, ROI = Republic of Ireland).

**Fig 4:**
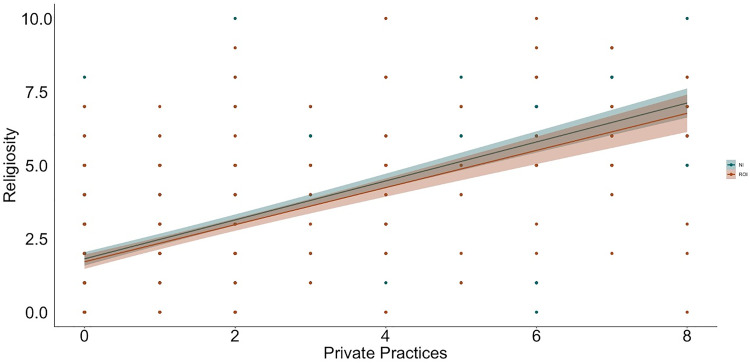
Relation between private practices and religiosity by context (NI = Northern Ireland, ROI = Republic of Ireland).

**Fig 5:**
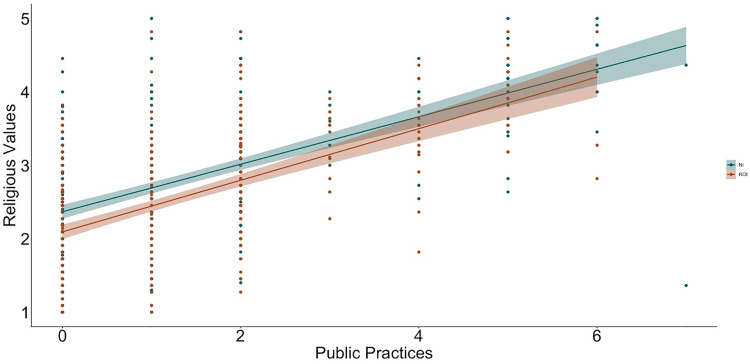
Relation between public practices and religious values by context (NI = Northern Ireland, ROI = Republic of Ireland).

**Fig 6:**
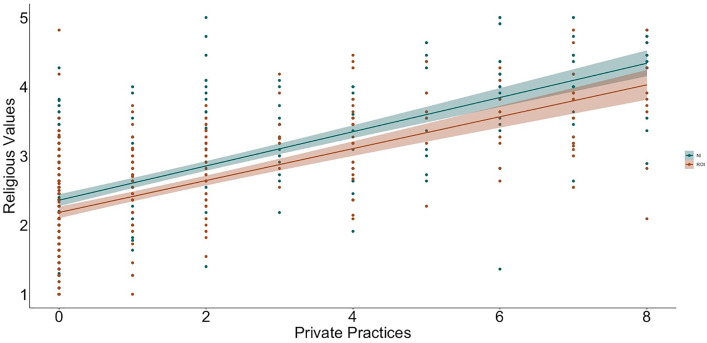
Relation between private practices and religious values by context (NI = Northern Ireland, ROI = Republic of Ireland).

**Fig 7:**
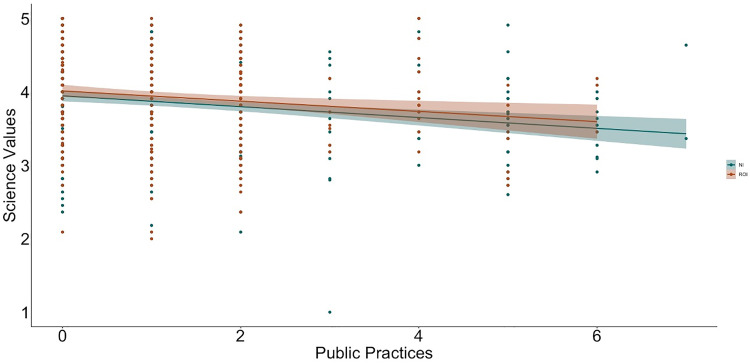
Relation between public practices and science values by context (NI = Northern Ireland, ROI = Republic of Ireland).

**Fig 8:**
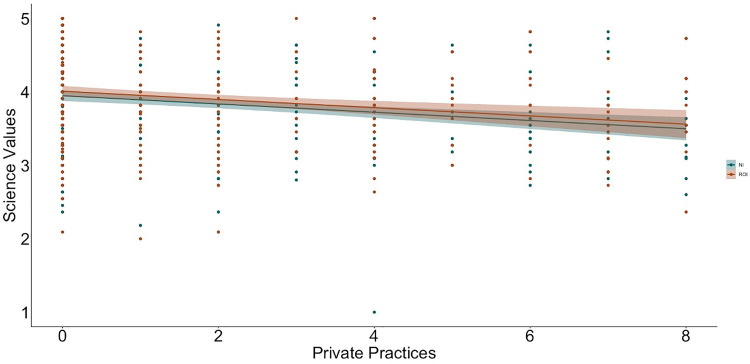
Relation between private practices and science values and by context (NI = Northern Ireland, ROI = Republic of Ireland).

**Fig 9:**
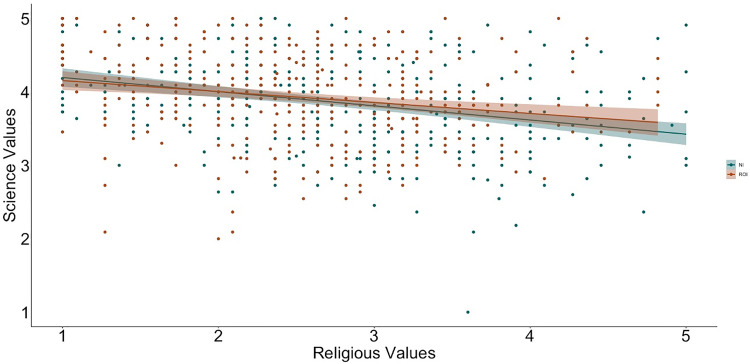
Relation between religious values and science values by context (NI = Northern Ireland, ROI = Republic of Ireland).

To examine relations between religiosity, religious practices, science values, and religion values, we used hierarchical linear regression (Tables 2 and 3 show the bivariate correlations between variables; Figs 1–9 depict relations between religiosity, religious practices, religious values, and science values pictorially). We analyzed religious values and science values as separate dependent variables. For each, in the first model, we included demographic variables (i.e., gender identity, perceived socioeconomic status, and education), in the second we entered context (dummy coded: Northern Ireland = 0, Republic of Ireland = 1), in the third we included religiosity (self-perceived, public practices, and private practices), in the fourth we included the other type of values (i.e., for the religious values we included science values as a predictor; for the science values we included religious values as a predictor). We initially included a fifth step with Context x Religiosity, Context x Public practices, Context x Private practices and Context x Values interactions but removed them as they did not increase the amount of variance explained (*F*s < 0.43, *p*s >.786).

**Table 2:** Bivariate correlations for key variables for whole sample.

	Context	Gender	Education	Perceived SES	Religiosity	Practices Public	Practice Private	Religious Values	Science Values
Context	1								
Gender	.09^*^	1							
Education	.21***	.04	1						
Perceived SES	.15***	−.06	.33***	1					
Religiosity	−.08^*^	.05	−.03	.01	1				
Practices Public	−.05	.06	.10**	.13***	.55***	1			
Practices Private	−.07^*^	.06	.003	.01	.63***	.66***	1		
Religious Values	−.16***	.01	−.05	−.01	.66***	.58***	.64***	1	
Science Values	.07	−.12***	.17***	.11**	−.29***	−.19***	−.22***	−.26***	1

*p <.05 ** *p* <.01 ***p <.001; Context (0 = Northern Ireland, 1 = Republic of Ireland); Gender (0 = Male, 1 = Female).

**Table 3:** Bivariate correlations for key variables by region.

	Gender	Education	Perceived SES	Religiosity	Practices Public	Practice Private	Religious Values	Science Values
Gender	1	.003	−.08	.01	.07	.04	.04	−.14^*^
Education	.04	1	.34***	.02	.06	−.06	−.03	.07
Perceived SES	−.08	.29***	1	.05	.18***	.02	.02	.07
Religiosity	.09	−.05	−.01	1	.53***	.59***	.63***	−.24***
Practices Public	.07	.15**	.12^*^	.57***	1	.61***	.56***	−.16**
Practices Private	.09	.07	.03	.66***	.70***	1	.61***	−.21***
Religious Values	.02	−.0001	.01	.67***	.60***	.66***	1	−.21***
Science Values	−.12^*^	.24***	.12^*^	−.32***	−.21***	−.23***	−.30***	1

*p <.05 ** *p* <.01 ***p <.001. Northern Ireland below the diagonal and the Republic of Ireland above the diagonal; Context (0 = Northern Ireland, 1 = Republic of Ireland); Gender (0 = Male, 1 = Female).

With religious values as the dependent variable (Table 4; 9 participants were removed because they did not have complete data for all variables, *N* = 730), the initial model with demographic variables did not add variance beyond the null model (*F*[3, 726] = 1.74, *p* =.157). Adding context to the model increased the amount of variance explained (*F*[1, 725] = 38.31, *p* <.001). Participants in Northern Ireland valued religion more than participants in the Republic of Ireland. Adding religiosity measures in the third model (*F*[1, 722] = 276.40, *p* <.001) and science values in the fourth model (*F*[1, 721] = 4.66, *p* =.031) also increased explained variance. Participants who reported being more religious, practiced religion more publicly or privately, valued religion more, or participants who valued science less, valued religion more.

**Table 4:** Results of regression analysis predicting religious values.

	Model 1			Model 2			Model 3			Model 4		
	*b*	SE	*t*	*b*	SE	*t*	*b*	SE	*t*	*b*	SE	*t*
Intercept	2.90***	0.19	15.09	2.84***	0.19	14.90	2.29***	0.13	16.97	2.60***	0.20	13.29
Gender	0.03	0.07	0.48	0.06	0.07	0.87	−0.04	0.05	−0.98	−0.06	0.05	−1.21
Education	−0.06	0.04	−1.47	−0.03	0.04	−0.75	−0.03	0.03	−1.12	−0.02	0.03	−0.80
Perceived SES	0.01	0.02	0.22	0.01	0.02	0.62	−0.01	0.02	−0.85	−0.01	0.02	−0.72
Context				−0.29***	0.07	−4.23	−0.19***	0.05	−3.64	−0.15***	0.05	−3.31
Religiosity							0.13***	0.01	10.90	0.13***	0.01	10.41
Practices Public							0.12***	0.02	5.79	0.12***	0.02	5.67
Practices Private							0.10***	0.01	7.37	0.10***	0.01	7.33
Science Values										−0.09^*^	0.04	−2.16
*Model F*	0.79			5.08***			124.50***			110.10		
*R* ^*2*^	<.01			.03			.55			.55		

*p <.05 ** *p* <.01 ***p <.001; Context (0 = Northern Ireland, 1 = Republic of Ireland); Gender (0 = Male, 1 = Female).

With science values as the dependent variable (Table 5; 9 participants were removed because they did not have complete data for all variables, *N* = 730), model two, which included demographic data resulted in an increase in variance explained beyond the null model (*F*[3, 726] = 12.72, *p* <.001). Participants who identified as male and those with higher educational attainment reported valuing science more. Whereas the inclusion of context in model three did not result in a significant increase in variance explained (*F*[1, 725] = 1.02, *p* =.313), adding religiosity and religious practices in the fourth model (*F*[1, 722] = 22.32, *p* <.001) as well as religious values (*F*[1, 721] = 4.66, *p* =.031) in the fifth model did. Participants higher in religiosity valued science less (practices were not significant predictors of science values), and, as with the previous model, those who valued religion more reported valuing science less.

**Table 5:** Results of regression analysis predicting science values.

	Model 1			Model 2			Model 3			Model 4		
	*b*	SE	*t*	*b*	SE	*t*	*b*	SE	*t*	*b*	SE	*t*
Intercept	3.36***	0.13	26.70	3.37***	0.13	26.69	3.54***	0.13	28.71	3.71***	0.14	25.12
Gender	−0.14***	0.04	−3.31	−0.15***	0.04	−3.38	−0.12**	0.04	−2.94	−0.13**	0.04	−3.02
Education	0.10***	0.02	4.09	0.10***	0.03	3.87	0.09***	0.02	3.94	0.09***	0.02	3.86
Perceived SES	0.02	0.02	1.32	0.02	0.02	1.22	0.03	0.01	1.74	0.02	0.01	1.68
Context				0.04	0.04	0.97	0.01	0.04	0.23	−0.001	0.04	−0.03
Religiosity							−0.05***	0.01	−4.60	−0.04***	0.01	−3.46
Practices Public							−0.03	0.02	−1.52	−0.02	0.02	−1.03
Practice Private							−0.01	0.01	−0.70	−0.001	0.01	−0.10
Religious Values										−0.07^*^	0.03	−2.16
*Model F*	11.67***			8.99***			15.13***			13.89***		
*R* ^*2*^	.05			.05			.13			0.13		

*p <.05 ** *p* <.01 ***p <.001; Context (0 = Northern Ireland, 1 = Republic of Ireland); Gender (0 = Male, 1 = Female).

Although the interactions containing context were not significant, we still explored whether the same results held in Northern Ireland and the Republic of Ireland by running separate regression models for each context (this analysis was not pre-registered). When we examined the data separately for Northern Ireland and the Republic of Ireland, we documented slightly different results. First, with valuing religion as the dependent variable, in Northern Ireland we found results consistent with the overall model (see Table 6): Valuing religion was positively related to religiosity as well as religious practices and it was negatively related to science values. In the Republic of Ireland, however, whereas valuing religion was positively related to religiosity and religious practices, it was unrelated to valuing science. When valuing science was dependent variable, we again found that the findings from Northern Ireland aligned with the overall model (see Table 7): valuing science was negatively related to religiosity and valuing religion. The pattern in the Republic of Ireland again diverged. Valuing science was negatively related to religiosity but was unrelated to religious values. Note that the lack of interaction and the fact that we did not pre-register these analyses requires that we interpret any differences between Northern Ireland and the Republic of Ireland with caution.

**Table 6:** Results of regression analysis predicting religious values – by context.

Northern Ireland									
	Model 1			Model 2			Model 3		
	*b*	SE	*t*	*b*	SE	*t*	*b*	SE	*t*
Intercept	2.80***	0.26	10.83	2.26***	0.18	12.40	2.70***	0.27	10.11
Gender (Male = 0)	0.04	0.10	0.46	−0.10	0.06	−1.53	−0.11	0.06	−1.74
Education	−0.02	0.05	−0.31	−0.03	0.03	−0.81	−0.01	0.04	−0.29
Perceived SES	0.01	0.03	0.38	−0.01	0.02	−0.35	−0.004	0.02	−0.19
Religiosity				0.14***	0.02	7.93	0.13***	0.02	7.45
Practices Public				0.11***	.03	4.08	0.10***	0.03	3.89
Practices Private				0.10***	0.02	5.13	0.10***	0.02	5.14
Science Values							−0.13^*^	0.06	−2.23
*Model F*	0.12			78.27***		68.49	65.20***		
*R* ^*2*^	<.01			.55			.56		
**Republic of Ireland**									
	**Model 1**			**Model 2**			**Model 3**		
	***b***	**SE**	***t***	***b***	**SE**	***t***	***b***	**SE**	***t***
Intercept	2.63***	0.30	8.72	2.13***	0.22	9.85	2.29***	0.30	7.52
Gender (Male = 0)	0.07	0.09	0.80	0.01	0.06	0.20	0.01	0.07	0.92
Education	−0.05	0.06	−0.86	−0.02	0.04	−0.59	−0.02	0.04	−0.56
Perceived SES	0.02	0.03	0.55	−0.02	0.02	−0.95	−0.02	0.02	−0.90
Religiosity				0.13***	0.02	7.41	0.12***	0.02	7.22
Practices Public				0.13***	0.03	4.02	0.13***	0.03	5.15
Practices Private				0.10***	0.02	5.20	0.10***	0.02	5.15
Science Values							−0.04	0.06	−0.75
*Model F*	0.45			59.15***			50.71***		
*R* ^*2*^	<.01			.51			.51		

*p <.05 ** *p* <.01 ***p <.001; Context (0 = Northern Ireland, 1 = Republic of Ireland); Gender (0 = Male, 1 = Female).

**Table 7:** Results of regression analysis predicting science values – by context.

Northern Ireland									
	Model 1			Model 2			Model 3		
	*b*	SE	*t*	*b*	SE	*t*	*b*	SE	*t*
Intercept	3.18***	0.16	19.81	3.36***	0.16	20.94	3.58***	0.19	18.95
Gender (Male = 0)	−0.14^*^	0.06	−2.39	−0.10	0.06	−1.82	−0.11^*^	0.06	−2.00
Education	0.14***	0.03	4.30	0.14***	0.03	4.46	0.13***	0.03	4.38
Perceived SES	0.02	0.02	1.01	0.03	0.02	1.38	0.03	0.02	1.34
Religiosity				−0.06***	0.02	−3.55	−0.04^*^	0.02	−2.47
Public Practices				−0.04	0.02	−1.78	−0.03	0.02	−1.29
Private Practices				−0.003	0.02	−0.15	0.01	0.02	0.42
Religious Values							−0.10**	0.04	−2.23
*Model F*	9.77***			13.24***			12.18***		
*R* ^*2*^	.07			.17			.18		
**Republic of Ireland**									
	**Model 1**			**Model 2**			**Model 3**		
	***b***	**SE**	***t***	***b***	**SE**	***t***	***b***	**SE**	***t***
Intercept	3.74***	0.21	17.39	3.88***	0.21	18.22	3.96***	0.24	16.40
Gender (Male = 0)	−0.16^*^	0.06	−2.43	−0.15^*^	0.06	−2.35	−0.15^*^	0.06	−2.34
Education	0.03	0.04	0.79	0.03	0.04	0.69	0.03	0.04	0.66
Perceived SES	0.02	0.02	0.87	0.03	0.02	1.17	0.03	0.02	1.13
Religiosity				−0.05***	0.02	−2.70	−0.04^*^	0.02	−2.22
Public Practices				−0.01	0.03	−0.29	−0.004	0.03	−0.12
Private Practices				−0.02	0.02	−1.07	−0.02	0.02	−0.83
Religious Values							−0.04	0.05	0.45
*Model F*	2.77^*^			5.19***			4.52***		
*R* ^*2*^	.02			.08			.09		

*p <.05 ** *p* <.01 ***p <.001; Context (0 = Northern Ireland, 1 = Republic of Ireland); Gender (0 = Male, 1 = Female).

Finally, to further understand the data we considered individual-level patterns (this analysis was not pre-registered). That is, although in Northern Ireland and the Republic of Ireland the overall pattern indicates that there is a negative relation between religion and science, it can be useful to consider the extent to which individual participants follow this overall pattern (see [28]). To explore such relations, we recoded the data so that participants who averaged over three on scale were considered “high” in that value and those who averaged below three on the scale were considered “low.” As can be seen in Table 8, the majority of participants in Northern Ireland (57%) and Republic of Ireland (65%) followed the pattern of the group (i.e., showed a pattern that indicated conflict between religion and science). Still, it is worth noting that the next largest proportion of participants in Northern Ireland (33%) and Republic of Ireland (24%) showed what could be described as co-existence (i.e., high in both religion and science).

**Table 8:** Percentage of participants who followed each data pattern.

	Science High-Religion High (Co-Existence)	Science High-Religion Low (Conflict)	Science Low-Religion High (Conflict)	Science Low-Religion Low (Rejection)	All other Patterns
Northern Ireland	33%	53%	4%	2%	7%
Republic of Ireland	24%	64%	1%	5%	5%

Note. Percentages do not add to 100% due to rounding. Two participants in Northern Ireland were removed because they did not have enough data for this comparison.

## Discussion

We investigated the relations between religiosity, religious values, and science values. Participants who were more religious or who valued religion more tended to devalue science. These findings held both in Northern Ireland, a post-accord context where religion remains closely tied to political identity and social division, as well as in the Republic of Ireland where there is no recent history of ethno-religious conflict. Still, exploratory analyses suggested the negative relation between religion and science may be more robust in Northern Ireland than in the Republic of Ireland. At the same time, in both contexts there are individuals who show a pattern more consistent with valuing both religion and science. We consider what these findings suggest about the religion-science connection, including considerations of limitations and future directions.

Prior research has provided evidence for both negative, positive, and null relations between religion and science (e.g., [8,10]). Thus, the link appears dependent on how it is conceptualized and tested, as well as the context in which it is assessed. We further tested the claim that societies with religion as more embedded may result in a weaker (negative) link between religion and science as suggested by Payir and colleagues [3]. The findings from our study, however, are less consistent with this specific assertion than we expected. That is, although Northern Ireland and the Republic of Ireland are societies where religion is highly embedded, our findings aligned more closely with the US and China, where there is a negative relation and a stronger dichotomy between religion and science. In contrast, they diverged from Iran, where religion is similarly embedded but the relation is null [3]. Importantly, we cannot directly compare our data with that of Payir and colleagues [3] as our methods differ slightly and the data were collected at different timepoints (i.e., [3] collected their data before the Covid-19 pandemic and our data were collected after the height of the pandemic), so it is possible that there are actually differences among these sites (i.e., US, China, Northern Ireland, and the Republic of Ireland). Future work comparing the relation between religion and science in the US, China, and Iran compared with Northern Ireland and the Republic of Ireland would help to clarify whether the impact of the Covid-19 pandemic masked important variability across these sites.

We also aimed to assess potential differences between Northern Ireland and the Republic of Ireland. Both societies have religion embedded in the formal education system (e.g., religious education is required). In Northern Ireland, however, religion also permeates social identity and power dynamics, whereas in the Republic of Ireland it is less present, in part due to the history of ethno-religious conflict in Northern Ireland. Accordingly, our aim was to explore whether such aspects of religious identity might lead to the devaluation of another domain (e.g., science). If this were the case, we might have expected the relation between religion and science to be weaker in the Republic of Ireland than in Northern Ireland. We did not find strong support for this possibility. Rather, we only documented that participants in Northern Ireland reported being more religious and valuing religion more than those in the Republic of Ireland. The valuation of science was equivalent across contexts. Moreover, the relation between religion and science was negative in both places. Though, follow-up exploratory analyses suggested that the relation exists between religiosity and science values in both locations, but valuing religion and valuing science were only negatively related in Northern Ireland after controlling for demographic variables and religiosity. These findings suggest that other similar features of the society (e.g., that religion and science are both embedded) may be more important for the relation than is a history of conflict and present-day power dynamics. Of course, in places where conflict is currently more prominent, or where the conflict is more about religiosity than ethno-religious identity then we might observe more of an effect. It is also possible that even within the contexts we studied here, that our results would have differed had we selected different samples from the population (e.g., more individuals higher in religiosity or more people who conduct science for a living).

It is worth considering that *how* we examined the relation between religion and science could have shaped our findings. When investigating religion and science, we chose to study values, but it would be equally interesting to consider beliefs. Although the negative relation between religious and science values was significant, the connection was only moderate in size. Perhaps, the negative link would be stronger in the context of beliefs or identity. That is, people may be able to value both religion and science more generally, but when it comes to specific beliefs (e.g., about where the world comes from or how humans came to be) they may be more likely to rely on one explanation more than another. Furthermore, our measures primarily assessed broad domain-level attitudes (e.g., perceived importance of science) rather than capturing multiple dimensions of trust in science and scientists. For example, frameworks that differentiate between trust in scientists’ competence, intentions, and specific domains (e.g., health, climate) could yield more nuanced insights (Krause et al., 2019) [29]. Future research could benefit from incorporating multidimensional measures of science valuation and trust (e.g., Nadelson et al., 2014) [30] to better capture the complexity of individuals’ perspectives.

On the other hand, it is also possible that the measures augmented the strength of the negative relation between religious and science values. Because we used parallel questions and these appeared together (science values followed by religious values) this format could have created a mutual exclusivity perspective in people’s minds which led to them feeling like they had to provide differentiated responses to the questions to appear coherent. We find this second possibility slightly less likely for two reasons. First, the same measure has previously been used to document a null relation in Iran [3], and our exploratory analyses on individual-level data suggest that a sizeable proportion of participants actually valued both religion and science simultaneously indicating that our measure did not “force” participants into a negative relation. Still, replicating these findings with distinct measures, especially taking a mixed-methods approach, would be valuable.

We also acknowledge limitations regarding the generalizability of our sample. Although Prolific allows access to a broad pool of participants, our sample was somewhat skewed toward individuals with higher levels of education and income. For example, 63% of our Northern Ireland participants reported having an undergraduate degree or higher, compared to 54% in a nationally representative sample (Northern Ireland Life and Times, 2023). In Republic of Ireland, 52% of our participants held a university degree, as compared to 42% in the national census (Census of Population, 2022). Our sample was also skewed toward higher income levels. In Northern Ireland, 28% of participants reported earnings in the top category on our income scale, whereas only 7% of the Northern Ireland Life and Times sample reported high income. In the Republic of Ireland, 32% of our participants reported incomes above the national median, whereas just 10% of the Survey on Income and Living Conditions Conditions (2023) sample fell within the top income decile. While income was measured on different scales across sources, this general pattern suggests our sample included a disproportionately high number of higher-income participants. These differences may limit the generalizability of our findings, particularly with regard to how religiosity and science values may vary across educational and socio-economic backgrounds. In both Northern Ireland and Republic of Ireland, access to religious education and exposure to scientific discourse are often shaped by class, and this sample bias should be considered when interpreting the broader applicability of our results.

Beyond methodological choices, there are other directions for future research. In the current manuscript, we explored the ground truth of the relation between religion and science values, but we are also interested in people’s beliefs about these relations. That is, do people expect more or less of a connection between religion and science than what has been documented in the literature and are these expectations also context dependent? Studying reasoning about the coexistence of beliefs could aid in our understanding of the polarization of religion and science across society. That is, even though our findings are consistent with the notion that religion and science are not entirely at odds, if the community believes that they are, political leaders and citizens alike may resist discussing issues that involve a perceived conflict between them. Examining the extent to which the religion-science link changes across age would also be valuable (Harris and Corriveau, 2021) [31]. As children are just learning about the world we may observe distinct relations, especially depending on when, from whom, and the context in which children learn about religion and science [32].

Our findings also have implications for understanding how the inter-relations between religion and science may vary across different policies, practices, and institutions in Northern Ireland and Republic of Ireland. For example, in locations where religion is firmly entrenched in political institutions, we might anticipate elevated tensions between valuation of science and religion, as compared with locations where secularization has separated religion from public institutions. These findings also highlight the importance of considering broader cultural and religious perspectives when interpreting an individual’s valuation of both science and religion. Such attention is important when determining the type of messaging that is most effective to present issues currently impacting the globe (e.g., climate change, vaccines). That is, given that in many contexts, there appears to be a negative relation between valuing religion and valuing science, it may be important for messengers (e.g., scientists, policy makers) to emphasize that any decision based in scientific evidence does not *require* opposing a religious perspective.

## Conclusions

In many societies, religion and science coexist. This concurrence, however, appears to come in the form of a negative correlation between the two on isle of Ireland. In other words, within these contexts, religion and science may operate in different spheres of society. This is especially interesting in this region of the world because religion and science both inform policies, such as educational curriculum. In conjunction with previous work (e.g., [8,3]), we highlight the necessity of studying religion and science around the world. Continuing this research, especially with an eye towards the links between identity, values, and beliefs could help to inform our understanding of polarization and conflict globally.

## Supporting information

S1 Table Pilot Study 1 and Pilot Study 2: Means [and 95% Cis] for the Science and Religious Values Survey in Northern Ireland
(DOCX)

## References

[1] Evans JH. Epistemological and Moral Conflict Between Religion and Science. Journal for the Scientific Study of Religion. 2011;50(4):707–27. doi: 10.1111/j.1468-5906.2011.01603.x

[2] Dixon T, Shapiro A. Science and religion: A very short introduction. Oxford University Press. 2022.

[3] Payir A, Davoodi T, Cui KY, Clegg JM, Harris PL, Corriveau K. Are high levels of religiosity inconsistent with a high valuation of science? Evidence from the United States, China and Iran. Int J Psychol. 2020;56(2):216–27. doi: 10.1002/ijop.12701 32617973

[4] Tech City. Tech nation 2017: At the forefront of global digital innovation. 2017. https://35z8e83m1ih83drye280o9d1-wpengine.netdna-ssl.com/wp-content/uploads/2018/04/Tech_City_2017_report_full_web.pdf

[5] Ecklund EH, Scheitle CP. Religion vs. science: What religious people really think. Social Forces. 2017;97:e4. doi: 10.1093/sf/soy048

[6] Gross N, Simmons S. The Religiosity of American College and University Professors. Sociology of Religion. 2009;70(2):101–29. doi: 10.1093/socrel/srp026

[7] Ecklund EH, Park JZ. Conflict Between Religion and Science Among Academic Scientists?. Scientific Study of Religion. 2009;48(2):276–92. doi: 10.1111/j.1468-5906.2009.01447.x

[8] Chan E. Are the religious suspicious of science? Investigating religiosity, religious context, and orientations towards science. Public Underst Sci. 2018;27(8):967–84. doi: 10.1177/0963662518781231 29874969

[9] Shtulman A. When competing explanations converge: Coronavirus as a case study for why scientific explanations coexist with folk explanations. In: Schupbach JN, Glass DH. Conjunctive explanations: The nature, epistemology, and psychology of explanatory multiplicity. Routledge. 2023.

[10] Legare CH, Evans EM, Rosengren KS, Harris PL. The coexistence of natural and supernatural explanations across cultures and development. Child Dev. 2012;83(3):779–93. doi: 10.1111/j.1467-8624.2012.01743.x 22417318

[11] Legare CH, Gelman SA. Bewitchment, biology, or both: the co-existence of natural and supernatural explanatory frameworks across development. Cogn Sci. 2008;32(4):607–42. doi: 10.1080/03640210802066766 21635349

[12] Menendez D, Hernandez IG, Rosengren KS. Children’s Emerging Understanding of Death. Child Dev Perspectives. 2020;14(1):55–60. doi: 10.1111/cdep.12357

[13] Watson-Jones RE, Busch JTA, Harris PL, Legare CH. Does the Body Survive Death? Cultural Variation in Beliefs About Life Everlasting. Cogn Sci. 2017;41 Suppl 3(Suppl 3):455–76. doi: 10.1111/cogs.12430 27859566 PMC10676006

[14] Shtulman A. Epistemic similarities between students’ scientific and supernatural beliefs. Journal of Educational Psychology. 2013;105(1):199–212. doi: 10.1037/a0030282

[15] Scheitle CP. U.S. College Students’ Perception of Religion and Science: Conflict, Collaboration, or Independence? A Research Note. Journal for the Scientific Study of Religion. 2011;50(1):175–86. doi: 10.1111/j.1468-5906.2010.01558.x

[16] Coyles D, Hamber B, Grant A. Hidden barriers and divisive architecture: The role of “everyday space” in conflict and peacebuilding in Belfast. Journal of Urban Affairs. 2023;45(6):1057–80. doi: 10.1080/07352166.2021.1930017

[17] ARK. Northern Ireland Life and Times Survey. ARK. 2008. http://www.ark.ac.uk/nilt

[18] ARK. Northern Ireland Life and Times Survey. 2020 [cited 2022 May]. http://www.ark.ac.uk/nilt

[19] O’Mahony E. Practice and belief among Catholics in the ROI. Irish Catholic Bishops’ Conference. 2011.

[20] Northern Ireland Department of Education. Northern Ireland curriculum. 2023. https://www.education-ni.gov.uk/publications/northern-ireland-curriculum

[21] Republic of Ireland Department of Education. Primary curriculum framework. 2023. https://www.gov.ie/en/publication/0db24-primary-curriculum-framework/

[22] Mitchell C. Religion, identity, and politics in Northern Ireland: Boundaries of belonging and belief. Routledge. 2016.

[23] Taylor LK, Dautel J, Rylander R. Symbols and labels: Children’s awareness of social categories in a divided society. J Community Psychol. 2020;48(5):1512–26. doi: 10.1002/jcop.22344 32176326

[24] Ganiel G. Pulpit to Public: Church Leaders on a Post-Brexit Island. Irish Studies in International Affairs. 2021;32(2):561–88. doi: 10.1353/isia.2021.0061

[25] Tonge J, Brannif M, Hennessey T, McAuley JW, Whiting S. The democratic unionist party: From protest to power. UOP Oxford. 2014.

[26] Erasmus. The clerical roots of the Democratic Unionist Party: Pious kingmakers in Northern Ireland. The Economist. 2017.

[27] Ganiel, G. (2016). Transforming post-Catholic Ireland: Religious practice in late modernity. Oxford University Press.

[28] McManus RM, Young L, Sweetman J. Psychology Is a Property of Persons, Not Averages or Distributions: Confronting the Group-to-Person Generalizability Problem in Experimental Psychology. Advances in Methods and Practices in Psychological Science. 2023;6(3). doi: 10.1177/25152459231186615

[29] Krause NM, Brossard D, Scheufele DA, Xenos MA, Franke K. Trends—Americans’ trust in science and scientists. Public Opinion Quarterly, 2019;83:817–36. https://doi.org/10.1093/poqnfz041

[30] Nadelson L, Jorcyk C, Yang D, Jarratt Smith M, Matson S, Cornell K, Husting V. I just don't trust them: the development and validation of an assessment instrument to measure trust in science and scientists. School Science and Mathematics, 2014;114:76–86. https://doi.org/10.1111/ssm.12051

[31] Harris PL, Corriveau KH. Beliefs of children and adults in religious and scientific phenomena. Current Opinion in Psychology, 2021;40:20–23. https://doi.org/10.1016/j.copsyc.2020.08.003 10.1016/j.copsyc.2020.08.003 32877835

[32] Weisman K, Ghossainy ME, Williams AJ, Payir A, Lesage KA, Reyes-Jaquez B, et al. The development and diversity of religious cognition and behavior: Protocol for Wave 1 data collection with children and parents by the Developing Belief Network. PloS One. 2024;19:e0292755. doi: 10.1371/journal.pone.029275 PMC10923471 38457421

