# Supplementary material for: Religiosity, religion values, and science values in Northern Ireland and the Republic of Ireland
Source: PLoS One. 2025 Oct 8;20(10):e0331205. doi: 10.1371/journal.pone.0331205 (PMC12507241; doi:10.1371/journal.pone.0331205)
Supplement: S1 Table — Pilot Study 1 and Pilot Study 2: Means [and 95% Cis] for the Science and Religious Values Survey in Northern Ireland (DOCX) [file pone.0331205.s001.docx]

**S1 Pilot Studies 1 and 2**

**Method**

***Participants***

**Pilot Study 1.** In the first study, 105 adults living in Northern Ireland consented to participate. Within this sample, however, some participants skipped questions or stopped taking the survey partway through; for this reason, we clarify the sample size included for each analysis. The sample was 79% female and 21% male (n = 61). In terms of education, 58% had received an undergraduate or post-graduate degree (n = 66). Thirty-six percent of the sample was Protestant, 51% was Catholic, 3% reported belonging to both backgrounds, and 11% belonging to another background (n = 73).

**Pilot Study 2.** In the second study, we had 110 adults living in Northern Ireland consent to participate. Within this sample, again, some participants skipped questions or stopped taking the survey partway through; for this reason, we clarify the sample size included for each analysis. Seventy-two percent of the sample was female and 28% was male (n = 102). Sixty-five percent of participants had an undergraduate or post-graduate degree (n = 101). Twenty-four percent of the sample identified as Catholic and 73% of the sample was Protestant, 1% identified with both backgrounds, and 2% reported that they had a different background (n = 99).

**Procedure.** The data for both of these studies were collected as part of larger projects. After providing informed consent, participants completed measures that aimed to assess their religiosity as well as their scientific and religious values. All measures were completed via Qualtrics Survey Software (Qualtrics, 2023). The data for the first study were collected from January 2020 to May 2020 and the data for the second study were collected in July 2021. These studies were approved by the Ethics Committee at [BLINDED FOR REVIEW]: protocol numbers: [BLINDED FOR REVIEW] and [BLINDED FOR REVIEW].

***Religiosity.*** In Study 1, religiosity was measured by three variables. Participants were asked how frequently they attend religious services and how frequently they engage in private worship (response options: more than once a week, once a week, once a month, only on special holy days, once a year, less than once a year, or never, practically never). These items were then rescored so that participants received a 1 if they selected once a week or more and a 0 if they selected another option. Participants were also asked if they considered themselves a religious person (1 = religious person; 0 = not a religious person). We averaged across these items to create one metric of religiosity (Payir et al, 2020). In Study 2, we only had a measure of the frequency with which participants reported engaging in worship (more than once a week, once a week, once a month, only on special holy days, once a year, less than once a year, or never, practically never, I am not a religious person). This item was then rescored so that participants received a 1 if they selected once a week or more and a 0 if they selected another option.

***Valuation of science and religion.*** Following Payir et al. (2020), participants reported how much they agreed with 22 statements about their valuation of religion (11 items) and science (11 items) on a 5-point scale from “strongly disagree” (1) to “strongly agree” (5). Example items include (“To me, it is important to have a [scientific/religious] outlook in life”; “It is important for children to be raised with a [scientific/religious] outlook in life).^[[1]](#footnote-1)^ See *Table S1* for the full set of statements and descriptive data for both Study 1 and Study 2.

***Transparency***

We do not have ethical approval to post our data publicly for these first two studies, but the data are available upon request. Analysis code and materials are available on the Open Science Framework (ADD OSF LINK ONCE ALL UPLOADED). Neither Study 1 nor Study 2 were publicly pre-registered.

**Results and Discussion**

***Pilot Study 1***

Consistent with prior research in the US, China, and Iran (Payir et al., 2020), initial tests showed that participants’ responses to the science values items were internally consistent (Cronbach’s alpha = .85, 95% Confidence Interval [CI] [.81, .89]) as were their responses to the religious values items (Cronbach’s alpha = .92 95% CI[.89, .94]. On average, participants showed moderate levels of religiosity (*M* = .36, 95% CI [.27, .45], n = 77). Participants also reported valuing science (*M* = 3.78, 95% CI [3.67, 3.89], n = 90) more than religion (*M* = 3.05, 95% CI[2.86, 3.24], n = 76; *t*[75] = 5.80, *p* < .001, *d* = 0.66, n = 76). More pertinent to our key research question, we examined the relation between religiosity, science values, and religion values. We found a negative correlation between religion and science values (*r*[74] = -.34, 95% CI[-.53, -.12], *p* = .003) and between religiosity and science values *r*[75] = -.33, 95% CI[-.52, -.12], *p* = .003). Not surprisingly, we also found a strong positive correlation between religion values and religiosity (*r*[74] = .70, 95% CI[.57, .80], *p* < .001).

***Pilot Study 2***

Aligning with Study 1 and prior research in US, China, and Iran, preliminarily analyses revealed that participants’ responses to the science items were internally consistent (Cronbach’s alpha = .88, 95% CI[.84, .91]) as were their responses to the religious items (Cronbach’s alpha = .90 95% CI[.87, .93]. Twenty-five percent of the sample reported worshiping (publicly or privately) once or week or more (n = 100). Participants also reported valuing science (*M* = 3.91, 95% CI [3.79, 4.03]) more than religion (*M* = 2.97, 95% CI[2.80, 3.14]; *t*[101] = 8.39, *p* < .001, *d* = 0.83; n = 102). Unlike, Study 1, we did not find a significant relation between science values and religious values (*r*[100] = -.11, 95% CI[-.30, .08], *p* = .265; n = 101) or between science and religiosity (*r*[98] = -.12, 95% CI[-.31, .08], *p* = .244; n = 99). Consistent with Study 1, religion values and religiosity were correlated (*r*[98] = .73, 95% CI[.62, .81], *p* < .001; n = 99).

These two initial studies on the relation between religion and science values in Northern Ireland provided seemingly conflicting evidence. Study 1 suggested that participants who value religion, and who are more religious, tend to value science less. In contrast, Study 2 suggested that there may be no relation between science values and religion values or between science values and religiosity. When comparing relations it’s important to not only consider the qualitative conclusions (i.e., that Study 1 yielded significant results and study Study 2 resulted in non-significant results) but also to directly compare the correlations to each other. Indeed, when we compared correlations across Study 1 and Study 2, we did not find any differences (religion values and science values: *z* = -1.58, *p* = .114; religiosity and science values: *z* = -1.44, *p* = .150; religion values and religiosity: *z* = -0.40, *p* = .692). Thus, from these two studies it is difficult to draw any clear conclusions about the relations between religion and science in Northern Ireland. In Study 3, then, we conducted a well-powered study to examine the relations between religion and science in Northern Ireland. We further expanded on the above studies in two ways. We added an additional context, the Republic of Ireland to understand how religious conflict might shape these relations. To further understand how religious and science values may relate to what gets passed from person to person, we added a measure of ethno-religious cultural socialization.

| *Table S1*. *Pilot Study 1 and Pilot Study 2: Means [and 95% Cis] for the Science and Religious Values Survey in Northern Ireland* | | |
| --- | --- | --- |
|  | Study 1 | Study 2 |
| Item | Mean [95% CI] | Mean [95% CI] |
| To me, it is important to have a scientific outlook on life. | 3.88 [3.69, 4.07] | 3.88 [3.70, 4.06] |
| *It is not very important to visit a science museum regularly. | 3.27 [3.08, 3.46] | 3.55 [3.34, 3.67] |
| *It is not very helpful to discuss scientific matters with other adults. | 3.87 [3.70, 4.03] | 3.94 [3.75, 4.13] |
| *It is not very important to read and understand scientific texts. | 3.68 [3.50, 3.86] | 3.79 [3.60, 3.98] |
| It is important to be open to guidance of people with scientific expertise. | 4.00 [3.84, 4.16] | 4.18 [4.03, 4.33] |
| It is important for children to be raised with a scientific outlook on life. | 4.03 [3.87, 4.18] | 3.70 [3.50, 3.89] |
| *It is not very important for children to visit a science museum regularly with their parents. | 3.51 [3.30, 3.72] | 3.93 [3.77, 4.09] |
| *It is not very helpful to discuss scientific topics with children. | 4.12 [3.94, 4.31] | 3.79 [3.60, 3.99] |
| *It is not very important for children to read and understand scientific texts. | 3.81 [3.63, 3.99] | 4.19 [4.02, 4.36] |
| It is important for children be open to the guidance of people with scientific expertise. | 4.11 [3.95, 4.27] | 3.91 [3.72, 4.11] |
| I turn to science for answers to key questions in life. | 3.38 [3.14, 3.61] | 4.11 [3.98, 4.24] |
| **Science Valuation Score Average** | **3.78 [3.67, 3.89]** | **3.91 [3.79, 4.03]** |
| To me, it is important to have a religious outlook on life. | 2.95 [2.64, 3.25] | 2.64 [2.36, 2.92] |
| *It is not very important to visit a place of worship regularly. | 2.71 [2.42, 3.00] | 2.87 [2.62, 3.13] |
| *It is not very helpful to discuss religious matters with other adults. | 3.01 [2.80, 3.23] | 3.27 [3.05, 3.50] |
| *It is not very important to read and understand religious texts. | 3.07 [2.82, 3.31] | 2.97 [2.72, 3.22] |
| It is important to be open to the guidance of people with religious expertise. | 3.14 [2.92, 3.37] | 2.80 [2.58, 3.03] |
| It is important for children to be raised with a religious outlook on life | 3.12 [2.84, 3.41] | 2.80 [2.55, 3.05] |
| *It is not very important for children to visit a place of worship regularly with their parents. | 2.93 [2.65, 3.22] | 2.81 [2.58, 3.05] |
| *It is not very helpful to discuss religious matters with children. | 3.58 [3.35, 3.80] | 3.45 [3.22, 3.68] |
| *It is not very important for children to read and understand religious texts. | 3.18 [2.94, 3.42] | 3.01 [2.79, 3.23] |
| It is important for children be open to the guidance of people with religious expertise. | 3.21 [2.96, 3.45] | 3.06 [2.84, 3.28] |
| I turn to religion for answers to key questions in life. | 2.70 [2.40, 3.00] | Not Included |
| **Religious Valuation Score Average** | **3.05 [2.86, 3.24]** | **2.97 [2.80, 3.14]** |

1. In Study 2, we were missing one question for the religion values questionnaire. [↑](#footnote-ref-1)
